# Secretion Systems in Gram-Negative Bacterial Fish Pathogens

**DOI:** 10.3389/fmicb.2021.782673

**Published:** 2021-12-15

**Authors:** Sophanit Mekasha, Dirk Linke

**Affiliations:** Section for Genetics and Evolutionary Biology, Department of Biosciences, University of Oslo, Oslo, Norway

**Keywords:** fish pathogen, Gram-negative, fish disease, secretion system, virulence factor, aquaculture

## Abstract

Bacterial fish pathogens are one of the key challenges in the aquaculture industry, one of the fast-growing industries worldwide. These pathogens rely on arsenal of virulence factors such as toxins, adhesins, effectors and enzymes to promote colonization and infection. Translocation of virulence factors across the membrane to either the extracellular environment or directly into the host cells is performed by single or multiple dedicated secretion systems. These secretion systems are often key to the infection process. They can range from simple single-protein systems to complex injection needles made from dozens of subunits. Here, we review the different types of secretion systems in Gram-negative bacterial fish pathogens and describe their putative roles in pathogenicity. We find that the available information is fragmented and often descriptive, and hope that our overview will help researchers to more systematically learn from the similarities and differences between the virulence factors and secretion systems of the fish-pathogenic species described here.

## Introduction

Production of cultured fish is one of the fastest-growing sectors of the aquaculture industries. The annual report from the Food and Agriculture Organization of the United Nations indicate that the production reached 179 million tons in 2018 (FAO, 2020). Sustainable production of farmed fish and their commercialization are primarily challenged by the expansion of infectious diseases caused by pathogenic microbes. Bacterial fish pathogens can cause systemic infection where they infect different organs of the fish, or they cause external infections by ulcerating the skin, gills, fin rots and mouth (Bernoth et al., 1997; Mohanty and Sahoo, 2007; Austin and Austin, 2016; Gourzioti et al., 2016; Ina-Salwany et al., 2019). In both cases, the fish cannot be marketed even in cases where the disease is not lethal. Thus, the high global spread of fish diseases causes great economic loss to the aquaculture industry and development of systematic prevention mechanisms is key to its sustainability.

Fish pathogens can infect both edible and ornamental fish species. Some ornamental fish such as zebrafish are used as host model organisms to understand the virulence mechanisms of pathogens in fish in general (Rowe et al., 2014; Nag et al., 2020). This review mainly covers virulence mechanisms of Gram-negative pathogens that target common edible fish. We have also made an attempt to differentiate the virulence factors that are proven to be involved in fish disease from the ones where the evidence is indirect, e.g., inferred from other host examples, and that thus need further investigation.

**FIGURE 1:**
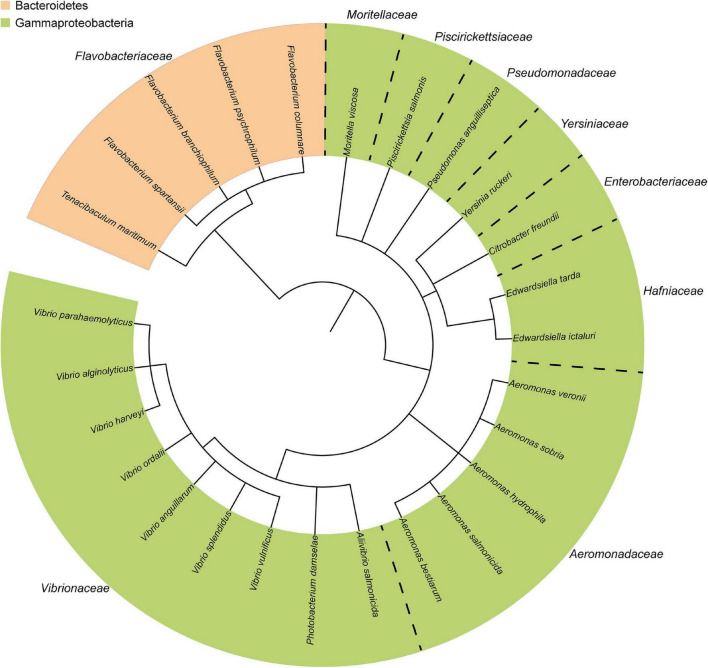
Taxonomic distribution of the most common Gram-negative bacterial fish pathogens. The phylogenetic tree was generated using the phyloT online tool (http://phylot.biobyte.de/), based on annotations from ITOL (http://itol.embl.de/) (Letunic and Bork, 2006).

The composition of the fish microbiota and the presence of pathogens has been evaluated by culture-dependent and/or advanced “omics” techniques for several years. The most prominent Gram-negative bacterial fish pathogens are distributed across the phyla Proteobacteria and Bacteroidetes (Figure 1), and a high number of identified and widely studied pathogens belong to the phylum Proteobacteria. The range of diseases found in fish reflect the diversity of virulence factors and virulence mechanisms utilized by these microbes. In general, bacterial infection is successful when the pathogen can successfully adhere to the host tissue, multiply and invade.

Information obtained from genome sequenced-bacterial strains shows that pathogenic and non-pathogenic fish-associated microbes can be very closely related [Figure 1, (Sudheesh et al., 2012)]. Thus, pathogens often acquire unique strategical adaptations specific for their infectious lifestyle that distinguishes them from their close, non-infectious relatives. Such adaptation is often associated with horizontal transfer of gene clusters of virulence genes located on either the chromosome (Naka et al., 2013; Amaro et al., 2015) or the more “flexible” gene pool of plasmids (Naka et al., 2011; Rivas et al., 2011; Amaro et al., 2015). Such blocks of virulence-associated DNA are known as “pathogenicity islands” (PAIs) that promote the pathogenicity of the strains (Hacker and Kaper, 1999; Hacker and Carniel, 2001; Osorio et al., 2015). Loss of potent virulence factors such as secretion systems either reduce or halt bacterial pathogenicity (Stanley et al., 2003; Dacanay et al., 2006; Fadl et al., 2006; Jyot et al., 2011).

Bacterial pathogens rely on the synergistic action of different virulence determinants and on specialized secretion systems to cause disease in susceptible hosts (Finlay and Falkow, 1997; Thanassi and Hultgren, 2000). Initiation of infection is often triggered by adherence of the pathogen to the skin or mucosal surface of the host tissue using attachment mechanisms such as non-fimbrial adhesins (Ostland et al., 1997; Weber et al., 2010; Guardiola et al., 2019), pili or fimbriae (Ho et al., 1990; Mattick, 2002; Gerlach and Hensel, 2007; Craig et al., 2019) which recognize specific receptors. A successful uptake of the pathogen into host cells is then mediated by specific invasion factors (e.g., invasins) which are either membrane anchored proteins of the pathogen or are secreted through specialized secretion systems. Invasins promote translocation of pathogens in host cell [(Meuskens et al., 2019), Yersinia spp.]. Once inside the host, the survival rate of pathogens is modulated by numerous structural and metabolic virulence factors such as capsules and iron acquisition systems, respectively (Lindler et al., 1998; Winkelmann et al., 2002; Hsieh et al., 2003; Møller et al., 2005; Guan et al., 2013; Balado et al., 2018). Encapsulated pathogens are resistant to phagocytosis due to the protective carbohydrate layer that blocks host immune response components from binding to immunogenic membrane proteins of the bacteria (Cress et al., 2014). In many infections, bacterial growth and metabolism is solely limited by the availability of free iron, which is typically completely sequestered in the host organisms. The competition for this limited resource forces pathogens to acquire and use high-affinity siderophore or heme-dependent iron acquisition systems to scavenge iron sequestered in host iron-binding proteins, such as transferrins or hemoglobin. Last but not least, the visible symptoms of disease such as inflammation, bleeding, or lethal shock is typically induced by different toxins. An important toxin is a structural component of Gram-negative pathogens, the lipopolysaccharides, that are a key component of the cell membrane and that exert intense biological effects on the host which may be lethal (Sampath, 2018). In addition to these so-called endotoxins, extracellular toxic proteins known as exotoxins, are produced and secreted by pathogens via specific secretion machinery. Unlike the extensive systemic damage of endotoxins that is mainly based on an adverse immune reaction, exotoxins typically target local tissues and are often restricted to particular cell types or receptors (Cavaillon, 2018). For other virulence factors, their identification and in-depth characterization is fundamental to the development of specific diagnostics and treatment tools. For instance, molecular, structural and biochemical characterizations of genes and proteins involved in secretion systems are useful for discovery of novel treatments for combating pathogenicity in bacteria (Costa et al., 2015; Alteri and Mobley, 2016).

In the case of fish pathogens, the knowledge on virulence factors and disease mechanisms is lagging behind, compared to the often very detailed information available for human pathogens. In this review, we summarize the current knowledge on one class of virulence determinants of fish pathogens, the secretion systems, and on the virulence factors that they secrete. In the course of collecting this information, we noticed that information on the Gram-positive fish pathogens shockingly sparse in this regard. This is why this review focuses on Gram-negative species, that cause most of the more notorious and economically relevant bacterial fish diseases. We hope that this review will be useful for researchers in the field of fish diseases, by providing an overview, and help in the quest to develop vaccines and treatments against fish pathogens that challenge the aquaculture industry worldwide.

### Bacterial Secretion Systems

Secretion systems are utilized by bacteria in processes such as growth, motility, pathogenic or symbiotic interactions with their hosts, and formation of microbial communities (Johnson et al., 2014; Costa et al., 2015; Gallique et al., 2017). In pathogenic species, many virulence factors are translocated across the membrane by dedicated secretion systems that are diverse in size, composition and architecture (Costa et al., 2015; Green and Mecsas, 2016).

Gram-negative bacteria possess two phospholipid membranes separated by a periplasmic space accommodating a thin layer of peptidoglycan chain. This creates additional challenges for protein secretion compared to the Gram-positive bacteria that contain only one lipid bilayer encircled by a peptidoglycan layer that forms a thick cell wall (Silhavy et al., 2010). In both Gram-positive and Gram-negative bacteria, two main secretion pathways employed for the secretion of proteins across the cytoplasmic membrane. These are the general secretory (Sec) pathway (Tsirigotaki et al., 2017) and the twin-arginine translocation (Tat) pathway (Palmer and Berks, 2012), where the first translocates unfolded proteins and the latter transports folded proteins. Thus, the two pathways translocate proteins into the periplasm in Gram-negative bacteria and are responsible for extracellular secretion of proteins across the single plasma membrane in Gram-positive bacteria (Palmer and Berks, 2012; Tsirigotaki et al., 2017). Outside of these two basic secretion pathways, both Gram-positive bacteria and Gram-negative bacteria possess diverse more specialized secretion systems (Green and Mecsas, 2016). It is worth noting that some species classified traditionally as Gram-positive bacteria such as *Mycobacteria* possess a complex additional membrane layer called the mycomembrane (Dulberger et al., 2020). These bacteria possess a specialized secretion system called type VII secretion system (T7SS) that translocates proteins across this complex cell envelope (Rivera-Calzada et al., 2021). The secretion systems of Gram-positive bacteria is out of the scope of the current review. Below, we list individual examples of Gram-negative fish-pathogenic species and their secretion systems and virulence factors, ordered by taxonomy (Table 1).

**TABLE 1:** List of common Gram-negative fish pathogens, including taxonomy information, disease type, virulence factors and virulence-related secretion systems obtained from experimental and/or genomic data.

Phylum	Strain	Disease	Virulence factors	Secretion systems (SS)	References
**Gram-negatives**					
Bacteroidetes	*Flavobacterium columnare*	Columnaris	Adhesins, gliding motility, Fe-acquisition systems, proteases, hemolysins chondroitin sulfate lyase	T9SS, T6SS^iii^	Boyd and Cornelis, 2001; Secades et al., 2001; Stringer-Roth et al., 2002; Kumru et al., 2017; Li et al., 2017; Kunttu et al., 2021
	*Flavobacterium psychrophilum*	Rainbow trout fry syndrome (RTFS) and cold water disease (CWD)	Adhesins, gliding motility, proteases, hemolysins elastase	T9SS	Ostland et al., 2000; Duchaud et al., 2007; Rochat et al., 2019; Barbier et al., 2020
	*Flavobacterium branchiophilum*	Gill disease	Adhesins, proteases, toxins	T9SS, T6SS^iii^	Ostland et al., 1997; Touchon et al., 2011; Kumru et al., 2020
	*Flavobacterium spartansii*	Pathological lesions, systemic infection	Adhesins, gliding motility, hemolysins toxins	T9SS, T6SS^iii^	Chen et al., 2017; Kumru et al., 2020
	*Tenacibaculum maritimum*	Tenacibaculosis	Adhesins, gliding motility, Fe-acquisition systems, chondroitin sulfate lyase	T9SS	Pérez-Pascual et al., 2017
Proteobacteria	*Vibrio anguillarum*	Vibriosis	Surface-hydrophobicity, Flagella, type IV pili, Fe-acquisition systems, metalloprotease, hemolysins	T2SS, T6SS	Rodkhum et al., 2005; Lopez and Crosa, 2007; Cianciotto and White, 2017
	*Vibrio harveyi*	Vibriosis	Flagella, non-pilus adhesins, Fe-acquisition systems	T3SS	Zane et al., 2014; Osorio, 2018; Zhang et al., 2021
	*Vibrio vulnificus*	Warm-water vibriosis	Flagella, Type IV pili, non-pilus adhesins, Fe-acquisition systems, protease, hemolysin, phospholipase	T1SS	Litwin and Byrne, 1998; Valiente et al., 2008; Jang et al., 2017; Guo et al., 2018
	*Vibrio parahaemolyticus*	Gastroenteritis, wound infections, and septicemia	Fe-acquisition systems, metalloprotease and serine protease, effectors	T2SS, T3SS*, T6SS*	Tanabe et al., 2003; Hiyoshi et al., 2010; Cianciotto and White, 2017; Li L. et al., 2019
	*V. alginolyticus*		Flagella, Fe-acquisition systems proteases, effectors	T3SS, T6SS	Cai et al., 2007; Wang Q. et al., 2007; Zhao et al., 2010; Salomon et al., 2015
	*V. ordalii*		Fe-acquisition systems	T3SS	Osorio et al., 2015; Ruiz et al., 2019
	*V. splendidus*		Flagella, Fe-acquisition systems non-pilus adhesins, effectors	T3SS	Song et al., 2018; Dai et al., 2020; Zhuang et al., 2020, 2021
	*Allivibrio salmonicida*	Cold-water vibriosis	Surface-hydrophobicity, Fe-acquisition systems, proteases, effectors	T3SS	Espelid et al., 1987; Winkelmann et al., 2002; Frey and Origgi, 2016
	*Photobacterium damselae*	Photobacteriosis, Skin ulcer	Fe-acquisition systems endopeptidase, toxin hemolysin, lipase, esterase-lipase, phospholipase	T2SS, T3SS	Labella et al., 2010; Rivas et al., 2011; Valderrama et al., 2019
Enterobacterials	*Yersinia ruckeri*	Enteric red mouth (ERM)	Type IV pilus, Fe-acquisition systems, proteases, toxins	T1SS, T4SS, T5SS	Fernández et al., 2002, 2004; Wrobel et al., 2018, 2019
	*Edwardsiella ictaruli*	Edwardsiellosis	Adhesin, Fe-acquisition system, proteases, effectors	T3SS	Thune et al., 2007; Abayneh et al., 2013; Abdelhamed et al., 2016; Castro et al., 2016
	*Edwardsiella tarda* (New name: *Edwardsiella piscicida*)	Edwardsiellosis	Adhesin, Fe-acquisition systems hemolysin, proteases, effectors	T3SS, T5SS, T6SS,	Wang X. et al., 2010; Jiao et al., 2010; Hu et al., 2016, 2019; Cheng et al., 2017
	*Citrobacter freundii*	Gastroenteritis and hemorrhagic septicemia	Adhesin, Fe-acquisition systems	T3SS	Leo et al., 2015; Pan et al., 2021
Aeromonadales	*Aeromonas bestiarum*	Motile Aeromonas septicemia (MAS)	Fe-acquisition systems,	T1SS, T2SS	Ali, 1996
	*Aeromonas salmonicida*	Furunculosis	Surface hydrophobicity, Fe-acquisition systems, hemolysin, lipase	T1SS, T2SS, T3SS*, T6SS	Titball and Munn, 1981; Burr et al., 2003; Dacanay et al., 2006; Reith et al., 2008; Balado et al., 2015
	*Aeromonas hydrophila*	Motile Aeromonas septicemia (MAS)	Surface hydrophobicity, adhesin, Fe-acquisition systems, hemolysins, proteases	T1SS, T2SS* T3SS, T6SS	Barghouthi et al., 1989b; Chopra et al., 1993; Fadl et al., 2006; Barger et al., 2020
	*Aeromonas veronii*	Motile Aeromonas septicemia (MAS)	Adhesin, Fe-acquisition systems	T3SS	Namba et al., 2008; Silver and Graf, 2009; Li et al., 2011; Tekedar et al., 2019b
	*Aeromonas sobria*	Motile Aeromonas septicemia (MAS)	Pili, hemolysin	T1SS, T2SS	Chakraborty et al., 1990
Thiotrichales	*Piscirickettsia salmonis*	Piscirickettsiosis	Adhesin, Fe-acquisition systems, chaperonin, toxins	T3SS, T4SS, T6SS	Rojas et al., 2013; Rozas and Enríquez, 2014; Ortiz-Severín et al., 2019
Alteromonadales	*Moritella viscosa*	Winter ulcer	Flagella, Type IV pili, hemolysin, peptidase	T1SS, T2SS, T6SS	Løvoll et al., 2009; Björnsdóttir, 2011; Bjornsdottir et al., 2012
Pseudomonadales	*Pseudomonas anguilliseptica*	“Red spot disease” also known as “sekiten-byo”	Flagella, capsular antigen	NA	Lönnström et al., 1994; Berthe et al., 1995; López-Romalde et al., 2003a

* Secretion systems where the direct involvement in fish disease still has to be established, as most data was generated with non-fish models. NA, data not available.

### Gram-Negative Bacteria

So far, eight types of Gram-negative bacteria secretion systems (designated as Type I-VI, Type VIII and Type IX or T1SS-T6SS, T8SS and T9SS) have been reported that are classified based on their composition, architecture, function and specificity. There is a multitude of reviews describing the functional and structural features of secretion systems (Gerlach and Hensel, 2007; Costa et al., 2015; Lasica et al., 2017; Burdette et al., 2018; Meuskens et al., 2019; Gorasia et al., 2020), allowing us to skip over the detailed description of each system, and to focus only on features that are relevant to fish pathogens. Extending the list of secretion systems, a recent report proposed the existence of a type X secretion system (T10SS) (Palmer et al., 2021). In short, these specialized secretion systems in Gram-negative bacteria can generally be classified into two categories. The first category includes those spanning both the inner and outer membranes such as the T1SS (Holland et al., 2005; Kanonenberg et al., 2018), T2SS (Nivaskumar and Francetic, 2014), T3SS (Deng et al., 2017), T4SS (Christie et al., 2014), T6SS (Russell et al., 2014a) and T9SS (Lasica et al., 2017; Veith et al., 2017; Gorasia et al., 2020). The second category includes the T5SSs (Meuskens et al., 2019) and T8SS (also known as the curli biogenesis system) (Bhoite et al., 2019; Yan et al., 2020) which span only the outer membrane (OM). This second category, and also the T2SS and T9SS rely on the Sec and Tat pathways to translocate their substrates into the periplasm, while the others are independent and secrete proteins directly from the cytosol into the medium or even into host cells in a single step.

## Bacteroidetes

The phylum Bacteroidetes, also known as Cytophaga-Flexibacter-Bacterioides (CFB), contains diverse anaerobe Gram-negative rods belonging to the *Flavobacteriaceae* family. Bacteroidetes inhabit diverse ecological niches including soil, sediments, oceans and freshwater and are the most predominant members of the animal gut microbiota. They are dominantly commensal and seldomly pathogenic to their host (Thomas et al., 2011; Fierer et al., 2012; An et al., 2013; Sunagawa, 2015; Hahnke et al., 2016).

### Flavobacteriaceae

The family *Flavobacteriaceae* is the largest family in the phylum Bacteroidetes, which contains more than ninety genera. This family includes important fish pathogens that belong to the genera *Flavobacterium* and *Tenacibaculum* (McBride, 2014). These pathogens cause severe diseases that impose significant economic losses in the aquaculture industry, and they are commonly isolated from diverse diseased finfish. Common fish pathogens of this family include *Flavobacterium columnare*, *F*. *psychrophilum*, *F*. *branchiophilum*, *F*. *spartansii* and *Tenacibaculum maritimum* (Wakabayashi et al., 1986; Bernardet and Bowman, 2006; Loch and Faisal, 2014; Chen et al., 2017).

*Flavobacterium columnare*, formerly known as *Flexibacter columnaris* (Bernardet et al., 1996) is an economically important fish pathogen. It is the causative agent of columnaris disease that results in skin lesions, fin erosion and gill necrosis, leading to a high degree of mortality (Declercq et al., 2013). *Flavobacterium psychrophilum*, also considered as an economically important bacteroidetes, causes severe mortality associated with hemorrhagic septicemia (Bernardet and Bowman, 2006). Disease terminologies such as “rainbow trout fry syndrome” (RTFS) and “cold water disease” (CWD) mostly denominate infections caused by *F. psychrophilum* and are based on the susceptibility of rainbow trout fry for these types of infection at temperatures below 10°C (Holt et al., 1989; Nematollahi et al., 2003b). *Flavobacterium branchiophilum* is a causative agent for gill disease, and while it is not usually isolated from internal organs, its severe mortality is associated with hemorrhagic septicemia. The known virulence factors related to this disease involve a cholera-like toxin and several adhesins [discussed below (Touchon et al., 2011)]. *Flavobacterium spartansii*, a recently discovered species that was initially isolated from salmon gills and kidneys causes systemic infection with symptoms such as muscle ulceration, necrotic gills, unilateral exophthalmia and friable kidneys (Loch and Faisal, 2014). *Tenacibaculum maritimum*, formerly known as *Flexibacter maritimus*, is the causative agent for tenacibaculosis, which is an ulcerative disease of marine fish that poses serious threats of economic losses in the aquaculture industry. Tenacibaculosis cause mostly external symptoms such as skin ulcers, mouth erosion, fin necrosis and rotted tails (Wakabayashi et al., 1986; Pérez-Pascual et al., 2017).

Despite the global challenge of the diseases and the financial losses caused by some members of *Flavobacteriaceae*, research into their mechanisms of pathogenicity is still in its infancy. However, recent studies revealed at least some of the virulence factors that these members of *Flavobacteriaceae* utilize to infect their fish host (Touchon et al., 2011; Chen et al., 2017; Li et al., 2017; Pérez-Pascual et al., 2017; Barbier et al., 2020; Kumru et al., 2020). Among these are genes and proteins responsible for gliding motility, various stress responses, adhesion, and they include secretion systems, degrading enzymes such as proteases, collagenases, polysaccharide lyases, sialidase and hemolysins that are utilized for the invasion and colonization of their host (Duchaud et al., 2007; Touchon et al., 2011; Tekedar et al., 2012; Chen et al., 2017; Pérez-Pascual et al., 2017; Kumru et al., 2020).

#### Transmission Routes and Adherence

Mucosal surfaces such as skin and gill are primary adhesion sites for several *Flavobacteriaceae* such a *F*. *columnare*, *F*. *branchiophilum*, *F*. *spartansii*, and *T. maritimum*. Several adhesin candidates have been identified, including surface lipopolysaccharides, capsules and fimbriae (Magarinos et al., 1995; Ostland et al., 1997; Klesius et al., 2008; Loch and Faisal, 2014; Pérez-Pascual et al., 2017). In addition, adherence based on hemagglutinins and lectin-like carbohydrates that are part of the capsule are responsible for attachment to gill tissue in *F. columnare* (Decostere et al., 1999; Bernardet and Bowman, 2006). *Flavobacterium psychrophilum* seem to adhere to gill tissue using several leucine-rich repeat proteins, similar to BspA and LrrA of bacteroidetes causing periodontal disease (Sharma et al., 1998; Kondo et al., 2002; Nematollahi et al., 2003a). *Flavobacterium psychrophilum* adheres to stomach and intestinal mucosa in rainbow trout (Nikoskelainen et al., 2001). In both *F. psychrophilum* and *F. columnare* the ability to agglutinate, adhere to, and hemolyze rainbow trout erythrocytes is associated with a sialic acid-binding lectin found in some serotypes of the pathogen (Lorenzen et al., 1997; Møller et al., 2003). The adherence of *F*. *branchiophilum* on mucosal surfaces of gills is the initial stage of infection in gill disease and this may be facilitated by several adhesin genes horizontally acquired by the pathogen (Ostland et al., 1997; Touchon et al., 2011).

Several adhesins of members of the *Flavobacteriaceae* family are secreted through T9SSs (Touchon et al., 2011; Pérez-Pascual et al., 2017; Barbier et al., 2020; Kunttu et al., 2021). *Flavobacterium columnare*, *F. psychrophilum*, *F. spartansii*, *T. maritimum*, possess genes involved in gliding motility, an active process independent of pili or flagella (Perry, 1973; Alvarez et al., 2006; Touchon et al., 2011; Loch and Faisal, 2014; Pérez-Pascual et al., 2017; Penttinen et al., 2018). *Flavobacterium columnare*, *F. spartansii, T. maritimum* and *F*. *psychrophilum* contain T9SS-associated genes such as *gld* and *spr*, that are involved in gliding motility. It has been shown that deletion of *gld* genes such as *gldD*, *gldG* and *gldN* not only leads to deficits regarding the ability of gliding motility and adhesion, but also negatively affects biofilm formation and extracellular hemolytic and proteolytic activities, indicating that T9SSs are involved in both gliding motility and secretion of proteins (Sato et al., 2010; Touchon et al., 2011; McBride and Nakane, 2015; Chen et al., 2017; Li et al., 2017; Pérez-Pascual et al., 2017; Barbier et al., 2020; Kumru et al., 2020). Despite the unique gliding motility properties of numerous bacteroidetes, some member species of the family such as *F. branchiophilum*, *T. maritimum* are reported to utilize a non-gliding motility mechanism as they contain pili, fimbriae or pili-like structures on their cell surface (Heo et al., 1990; Rahman et al., 2014).

#### Survival Mechanisms in the Host

Pathogens have evolved different mechanisms to overcome bacterial competition in order to secure efficient colonization of their host. Among the bacteroidetes, that are the overall most dominant phylum of animal gut microbiota, *F*. *johnsoniae* is known to possess a unique T6SS named T6SS*^iii^* which is directly involved in bacterial competition (Russell et al., 2014b). Similarly, genomic analysis of some strains of *F*. *branchiophilum*, *F. spartansii*, and *F*. *columnare* show the presence of T6SS*^iii^* coding genes that might be involved in host bacterial competition (Abby et al., 2016; Kumru et al., 2017, 2020).

Furthermore, bacteroidetes evolved diverse survival mechanisms to escape the hostile environments generated by host cells to hinder their colonization. These include defense mechanisms against bacterial pathogens such as the production of reactive oxygen species (ROS) by host macrophages (Secombes, 1996). Bacteroidetes harbor genes encoding superoxide dismutase, catalase-peroxidase, and thiol peroxidase to resist ROS-mediated killing (Duchaud et al., 2007; Chen et al., 2017; Levipan et al., 2018). Iron acquisition systems have been identified in *F*. *columnare* (Guan et al., 2013; Zhang et al., 2017), *F*. *psychrophilum* (Møller et al., 2005), *F*. *spartansii* (Chen et al., 2017), *F*. *branchiophilum*, and *T*. *maritimum* (Avendaño-Herrera et al., 2005), whose function is to compete for the limited iron stock in host cells. The uptake of iron-siderophore complexes by bacteroidetes are not fully explored, however, several TonB-dependent outer membrane receptors such as, OmpA related proteins, ferrichrome-iron receptor precursor (FhuA) and ferric uptake receptor (Fur) protein are identified in the genomes of *F*. *columnare* (Dumpala et al., 2010; Guan et al., 2013), *F*. *psychrophilum* (Alvarez et al., 2008) *T*. *maritimum* (Avendaño-Herrera et al., 2005) that may function as iron importer across the outer membrane as is shown for several Gram-negative bacteria (Letain and Postle, 1997; Braun et al., 1998).

Secretion of exopolysaccharides by pathogenic bacteria provides a beneficial environment for survival in the host. *F. columnare* secretes large quantities of exopolysaccharides during infection using polysaccharide exporters that transport polysaccharides from the cytoplasm to the periplasm (Zhang et al., 2017).

#### Proteases and Their Secretion Systems

Fish-pathogenic flavobacteriaceae isolated from different fish species produce several secreted proteases with elastase, gelatinase, collagenase, and caseinase activities that may have significant roles in virulence (Bertolini et al., 1994; Dalsgaard and Madsen, 2000; Ostland et al., 2000; Duchaud et al., 2007). The two economically important bacteroidetes *F*. *psychrophilum* and *F*. *columnare* cause severe mortality associated with hemorrhagic septicemia and columnaris, respectively, by using multi-factorial virulence factors including the metalloproteases (Griffin, 1987; Secades et al., 2001, 2003). Fpp1 and Fpp2, which are calcium dependent proteases that cleave protein-rich extracellular matrices of connective and muscular tissues during colonization (Secades et al., 2001, 2003). Fpp1 and Fpp2 contain the conserved carboxy terminal domain (CTD-domain) for protein secretion through T9SS, which suggest that the T9SS is the secretion system for these metalloproteases (Li et al., 2017; Barbier et al., 2020).

Virulence-associated collagenases are among the conserved virulence factors in *F*. *spartansii* (Chen et al., 2017), *F. columnare* (Olivares-Fuster and Arias, 2008), *F*. *psychrophilum* (Nakayama et al., 2016), and *T*, *maritimum* (Pérez-Pascual et al., 2017). They are used by the pathogens to disrupt collagen-rich tissues of the host. The specific secretion pathways for these collagenases are yet to be explored, however, different studies show the dependency of secreted proteolytic enzymes on T9SS (Chen et al., 2017; Li et al., 2017; Pérez-Pascual et al., 2017).

Polysaccharide lyases such as Chondroitin AC lyase that degrade complex acidic polysaccharides such as hyaluronic acid and chondroitin sulfates located mainly on the extra-cellar matrix of fish tissues are potential virulence factors in *F*. *columnare*, where this has been shown *in vivo* using rainbow trout (Stringer-Roth et al., 2002; Li et al., 2017) and in *T*. *maritimum* where the activity was tested on chondroitin sulfate-supplemented agar (Pérez-Pascual et al., 2017). These enzymes are suggested to initiate and promote infection and are secreted by T9SS: mutational deactivation of a component of T9SS, gldN, in *F*. *columnare* lead to the loss of the ability to degrade chondroitin (Li et al., 2017; Pérez-Pascual et al., 2017).

Extracellular elastases produced by *F*. *psychrophilum* may have roles in the digestion of host tissues (Madsen and Dalsgaard, 1998; Nematollahi et al., 2003b; Rochat et al., 2019). Unlike for most other virulence factors of fish-pathogenic flavobacteriaceae the secretion pathway of elastase from *F. psychrophilum* is T9SS-independent (Rochat et al., 2019). Yet, the specific secretion system for the elastase is unidentified.

Glycoproteins are described to be involved in pathogenesis in several pathogenic Gram-negative bacteria (Guerry, 2007), taking part in processes such as cell adhesion and motility (Virji, 1997; Young et al., 2002; Grass et al., 2003; Szymanski et al., 2003). In *F*. *psychrophilum*, several glycoproteins were previously identified and predicted to promote infection (Merle et al., 2003; Dumetz et al., 2007). An outer membrane glycosyltransferase, FpgA, expressed by *F*. *psychrophilum* has been shown to promote polysaccharide-mediated gliding motility and bacteria-host cell interactions (Fernández-Gómez et al., 2013; Pérez-Pascual et al., 2015). Mutations in the *fpgA* gene not only impair pathogenicity, but also downregulate expression of the metalloproteases Fpp2 and Fpp1 discussed earlier in this section (Pérez-Pascual et al., 2015). However, neither the secretion apparatus for the metalloproteases Fpp1 and Fpp2 nor its connection to the enzyme FpgA itself is understood.

#### Hemolysins and Their Secretion Systems

Hemolysins are potent virulence factors for fish pathogens such as *F. psychrophilum*, *F. columnare*, *F. spartansii* and *T. maritimum.* They are involved in the lysis of host erythrocytes and in recovering their hemoglobin-bound iron pool for growth (Avendaño-Herrera et al., 2005; Møller et al., 2005; Högfors-Rönnholm and Wiklund, 2010; Chen et al., 2017; Kayansamruaj et al., 2017). Hemolytic enzymes play a part in the virulence of *F*. *psychrophilum* (Högfors-Rönnholm and Wiklund, 2010). *F. psychrophilum* contains a putative hemolysin gene, FP0063, with 53% similarity with the hemolysin of *Vibrio anguillarum* VAH5 (see the Vibrio section below). The Vibrio hemolysin has been shown to degrade erythrocytes creating a devastative host cell damage (Rodkhum et al., 2005; Duchaud et al., 2007). Similarly, *F*. *spartansii* harbors virulence-related hemolysin genes that are vital in pathogenesis process such as tissue damage and sepsis (Chen et al., 2017). *Flavobacterium columnare* contain hemolysin genes that are prominent for pathogenesis (Kayansamruaj et al., 2017; Kumru et al., 2017). *Tenacibaculum maritimum* contains a gene encoding for sphingomyelinase, a toxin directly involved in hemolysis (Oda et al., 2010; Pérez-Pascual et al., 2017). The secretion mechanisms of hemolysins of fish-pathogenic flavobacteriaceae is not yet clear. However, mutations affecting genome regions encoding T9SS responsible for gliding motility, including the examples GldJ, GldK, GldM, and GldN from *F. psychrophilum* led to reduced hemolytic activity, indicating that the secretion of hemolysins is at least indirectly influenced by T9SS (Castillo et al., 2015; Barbier et al., 2020).

#### Other Protein Toxins and Their Secretion Systems

*Flavobacterium spartansii* harbors genes encoding for toxin thiol-activated cytolysin (TACY) which possibly modulate or lyse the function of enterocyte membranes through formation of pore (Chen et al., 2017). *Flavobacterium branchiophilum* produces a cholera-like toxin protein, identified as FBFL15_0919 (Touchon et al., 2011), which has high sequence similarity with the cholera toxin (CTA) produced by *V*. *cholera* and the heat-labile toxin (LTA) produced by enterotoxigenic *Escherichia coli*. The LTA and CTA toxins stimulate adenylate cyclase and provoke the massive loss of fluid and electrolytes through the intestinal epithelium of the host (Sixma et al., 1991; de Haan and Hirst, 2000; Broeck et al., 2007). Similarly, FBFL15_0919 is speculated to disturb the osmoregulatory function of the epithelial cells of the gills. Gill cells are involved in excretion of urea and in uptake of salts, in addition to oxygen uptake (Wilkie, 2002). The exact secretion mechanism for FBFL15_0919 is not yet investigated, however, CTA is reported to be exported by T2SS (Robien et al., 2003).

Despite the availability of genome sequences of fish pathogens such as *F*. *psychrophilum*, many of the secretion systems for virulence factors that are key to the pathogenesis of other Proteobacteria are not yet described in detail in *Flavobacteriaceae*. Genes for key components for secretion systems from almost all classes have been found in the genomes of different *Flavobacterium* species, but have not yet been directly linked to virulence experimentally (Kumru et al., 2020). Much more is known about the components for T9SSs (Duchaud et al., 2007). Mutation of genes involved in gliding (the *gld* genes), which are components of the T9SS in *F*. *johnsonia* (a well-studied member of the fish-pathogenic bacteroidetes) lead not only deterioration of motility but also to significant changes in the secretion of extracellular enzymes (McBride et al., 2003; Braun et al., 2005; Braun and McBride, 2005) strongly suggesting that the T9SS may be the most crucial virulence factor of bacteroidetes. It facilitates pathogenicity through providing multiple virulence tools for successful infection ranging from colonization to host cell disruption processes (Li et al., 2017; Penttinen et al., 2018; Barbier et al., 2020).

## Proteobacteria (Class: Gammaproteobacteria)

### Vibrionales

The Vibrionales are a separate order within the Proteobacteria that contains only one Family, the *Vibrionacae*. They encompass many important facultative anaerobic fish pathogens, mainly from the genera *Vibrio, Allivibrio* and *Photobacterium*.

Vibrios are rod-shaped ubiquitous and important pathogens to varied marine and freshwater fish species (Rucker, 1959). Vibriosis, one of the common diseases in the aquaculture industry with outbreaks that lead to high mortality of farmed fish, imposes severe negative impacts on productivity, sustainability and profitability. Diverse pathogenic vibrio species have been isolated from diseased fish with vibriosis symptoms that include bruised, red spotted and ulcerated skin, mouth and fin sores, and systemic infection symptoms like tissue necrosis, hemorrhagic muscles, and other bleedings (Buller, 2004; Toranzo et al., 2005). All pathogenic vibrios, including the human-pathogenic *Vibrio cholerae*, are directly connected to aquatic environments. The genome of the vibrios is encompasses two chromosomes, an unusual feature compared to other bacterial genera (Okada et al., 2005). The most common pathogenic vibrio species responsible for fish vibriosis include *V*. *anguillarum*, *V*. *harveyi*, *V*. *vulnificus*, *V. parahaemolyticus*, *V*. *alginolyticus*, *V*. *ordalii*, *V*. *splendidus*, and in addition the species *Allivibrio salmonicida* and *Photobacterium damselae* that are part of closely related *Vibrionacae* genera (Austin B. et al., 2003; Austin and Austin, 2012). *Vibrio cholerae* is able to colonize diverse fish species indicating that fish could be a vector for *V. cholerae*. However, direct pathogenicity of *V. cholerae* to fish is debatable (Halpern and Izhaki, 2017).

#### Transmission Routes and Adherence

The main routes of transmission for vibriosis include direct contact (entry through skin and/or damaged mucous layers) and oral (ingestion) transmissions (Grisez et al., 1996; Svendsen and Bøgwald, 1997; Jun and Woo, 2003; Weber et al., 2010). The initial stage of infection involves colonization of the skin mucosa and biofilm formation facilitated by exopolysaccharides (Croxatto et al., 2007; Weber et al., 2010). Adherence of the bacteria to host surfaces and survival in the host are particularly determined by the surface hydrophobicity of pathogenic bacteria (Lee and Yii, 1996; Balebona et al., 1998). For instance, *V. anguillarum* and *V. salmonicida* utilize surface hydrophobicity to survival in hosts organism (Horne and Baxendale, 1983; Espelid et al., 1987; Hjelmeland et al., 1988).

*Vibrionaceae* utilize specific pili or various non-pilus adhesins that facilitate adhesion to host cells and initiate infection. Adhesion can be mediated by flagella in *V. anguillarum*, *V. alginolyticus*, *V. harveyi*, *V. splendidus*, and *V. vulnificus* indicating that motility is key for adhesion in these organisms (Milton et al., 1996; O’Toole et al., 1996; Ormonde et al., 2000; Luo et al., 2016; Dai et al., 2020; Xu et al., 2021). In addition to flagella, the outer membrane protein TolC and NADH oxidase dihydrolipoamide dehydrogenase play pivotal roles in the adhesion of *V. harveyi* and *V. splendidus*, respectively (Dai et al., 2019; Zhu et al., 2019). Experiments performed on non-fish cell lines show other factors including the adhesin *Vp*adF, enolase, capsular polysaccharide, T6SSs, multivalent adhesion molecule 7 (MAM7) and a mannose-sensitive hemagglutinin (MSHA) pilus bind to different receptors to facilitate cell attachment by *V. parahaemolyticus* (Hsieh et al., 2003; Krachler et al., 2011; Yu et al., 2012; O’Boyle et al., 2013; Jiang et al., 2014; Liu and Chen, 2015). A β-pore forming toxin, phobalysin, plays a pivotal role in host cell adherence in *Photobacterium damselae* (Rivas et al., 2015; von Hoven et al., 2018).

Survival mechanisms in the hostOnce inside the host, survival of *Vibrionaceae* is achieved through distinct virulence mechanisms such as regulation of iron acquisition, neutralization of acidic environments, combating oxidative stress, induction of host immune cell death, and immune evasion. *V. vulnificus* neutralizes acidic environments of the host gut and tolerates oxidative stress by converting lysine to cadaverine and CO_2_ using lysine decarboxylase and manganese superoxide dismutase (SOD) respectively (Rhee et al., 2002; Kim et al., 2005; Jones and Oliver, 2009). Invasion and inter-microbial competition of *V. cholerae* in the gut of zebrafish is mediated by a T6SS where the syringe-like system plays a role in intensifying the host gut contractions which lead to expulsion of resident microorganisms (Logan et al., 2018).

Several *Vibrionaceae* possess finely regulated and high-affinity siderophore or heme-dependent iron acquisition systems to compete for iron sequestered by iron-binding proteins such as transferrins or in heme in host organisms (Thode et al., 2018; Richard et al., 2019). Siderophore-dependent iron acquisition systems synthesize and secrete siderophores into the environment to chelate ferric iron. The secretion mechanisms for siderophores are not fully understood in the *Vibrionaceae* but a major facilitator superfamily protein (MFS)-mediated efflux pump seems to play an important role (Li and Ma, 2017). No hemophores to chelate iron from hemoproteins have yet been identified in fish-pathogenic species from the *Vibrionaceae* family (Wandersman and Delepelaire, 2004).

*Vibrio anguillarum*, *V. vulnificus*, *V. ordalii*, *V. harveyi*, *Photobacterium damselae*, *V. parahaemolyticus*, *V. alginolyticus*, *A. salmonicida*, and *V. splendidus* utilize siderophore-mediated iron acquisition systems. The uptake of both ferric-siderophore complex and heme across the membrane is performed by ABC transporters which utilize TonB complex as energy transducers (Stork et al., 2004; Thode et al., 2018).

Depending on the serotype, *V. anguillarum* produce three distinct iron-uptake systems: anguibactin and its outer membrane receptor FatA (Lopez and Crosa, 2007; López et al., 2007; Naka et al., 2013), piscibactin and its outer membrane receptor FrpA (Balado et al., 2018; Valderrama et al., 2019) and vanchrobactin and its outer membrane transport FvtA (Soengas et al., 2006, 2008; Balado et al., 2008, 2009). Like *Vibrio anguillarum*, *Photobacterium damselae* and *V. ordalii* produce the piscibactin siderophore where the receptor in the former is FrpA and in the latter is unclear (Souto et al., 2012; Ruiz et al., 2019; Valderrama et al., 2019). *Vibrio vulnificus* synthesize vulnibactin and its uptake is mediated by VuuA (Webster and Litwin, 2000; Kim et al., 2006; Alice et al., 2008). *Vibrio harveyi* produce amphi-enterobactin and its uptake receptor FapA (Zane et al., 2014; Naka et al., 2018). *Vibrio parahaemolyticus* produces the siderophore vibrioferrin and its receptor PvuA (Funahashi et al., 2002; Tanabe et al., 2003). Like *V. parahaemolyticus, V. alaginoluticus* contains gene clusters for vibrioferrin biosynthesis (Wang Q. et al., 2007). *Allivibrio salmonicida* utilize bisucaberin that is recognized by BitA (Winkelmann et al., 2002; Kadi et al., 2008). *Vibrio splendidus* utilize a hydroxamate-based siderophore and IutA is proposed as a potential receptor (Song et al., 2018). Besides the siderophore iron acquisition system, *V. vulnificus* directly use heme from hemoglobin as host iron sources, where the uptake of heme is facilitated by outer membrane protein HupA (Litwin and Byrne, 1998). *Vibrio anguillarum* possess a heme-utilizing system where two outer membrane proteins, HuvA and HuvS, perform heme uptake (Mazoy et al., 1996; Mazoy and Lemos, 1996; Mouriño et al., 2005), and where the complex HuvBCD is required for subsequent transport of periplasmic heme into the cytosol (Mouriño et al., 2004).

#### Proteases and Secretion Systems

Extracellular proteases are associated with the pathogenesis of several *Vibrionacae*. The extracellular zinc metalloproteases EmpA and PrtV of *V. anguillarum* are potent virulence factors with mucinase activities that are required for infecting the gastrointestinal tract of diverse fish species such as salmon, turbot and flounder (Norqvist et al., 1990; Milton et al., 1992; Denkin and Nelson, 1999, 2004; Zhao-lan et al., 2002; Mo et al., 2010; Frans et al., 2011). The secretion of EmpA and PrtV is mediated by a T2SS (Zhang et al., 2006; Rompikuntal et al., 2015). EmpA and PrtV belong to the M4 and M6 family of peptidases, respectively, and are translated in inactive form which passes through a maturation process involving proteolytic cleavages during secretion through the T2SS (Milton et al., 1992; Staroscik et al., 2005; Zhang et al., 2006; Varina et al., 2008; Mo et al., 2010; Rompikuntal et al., 2015). Besides its proteolytic activity, EmpA activates hemolysins promoting disruption of red blood cells in fish (Han et al., 2011). PrtV contains gelatinase, protease and glycosidase activity, and a *prtV* mutant of *V. anguillarum* strain show reduced infection, growth and hemolytic activity in turbot and turbot cell lines (Mo et al., 2010).

*Vibrio parahaemolyticus* produce multiple extracellular metalloproteases such as the tissue-degrading protease VPM, and the collagenases PrtV and VppC. In addition, serine proteases Vpp1/protease A with cytotoxic activity, VpSP37 with gelatinase activity, and PrtA with hemolytic and cytotoxic activity are produced as virulence factors (Osei-Adjei et al., 2018). Similarly, *V. alginolyticus* produce extracellular alkaline serine protease A and a VppC homolog where the former is an exotoxin and lethal to fish (Takeuchi et al., 1992). *Vibrio vulnificus* produce an extracellular metalloprotease Vvp, however, its involvement in virulence is debatable (Valiente et al., 2008). The secretion mechanisms of these metalloproteases are not yet described.

#### Hemolysins and Secretion Systems

Six pore-forming hemolysins, Vah1-5 and Rtx, are responsible for disrupting red blood cells leading to hemorrhagic septicemia in *V. anguillarum* (Hirono et al., 1996; Rodkhum et al., 2005). Vah1-5 belong to the HylA hemolysin family (Hirono et al., 1996; Zhang and Austin, 2005). Secretion factors of Vah1-5 from *V. anguillarum* are not documented; however, HylA hemolysins are generally known to be secreted via T1SS (Thomas et al., 2014). Like the metalloproteases EmpA and PrtV of *V. anguillarum*, hemolysins in the HylA family are translated as pre-proteins where the production of mature hemolysins requires multiple processing steps during secretion (Zhang and Austin, 2005). All Vah hemolysins show hemolytic activity on fish erythrocytes, with Vah4 displaying the strongest virulence effects (Rodkhum et al., 2005). RtxA (repeats-in-toxin-A), a multifunctional extracellular protein secreted by diverse Gram-negative fish pathogens, is responsible for cytotoxic and hemolytic attacks in fish (Li L. et al., 2008). The secretion of unfolded Rtxs is performed by a T1SS where folding is initiated only after passage (Linhartova et al., 2018). The expression and secretion of *V. vulnificus* RtxA1 toxin is regulated by a general stress response regulator, RpoS (Guo et al., 2018).

*Vibrio parahaemolyticus* possess two pathogenic hemolysins, thermostable direct hemolysin (TDH) and TDH-related hemolysin (TRH) (Li L. et al., 2019). *Vibrio parahaemolyticus* possess multiple secretion systems, a T2SS and two T3SSs (T3SS1 and T3SS2) where the T2SS and T3SS2 export the hemolysins. TDH possesses exotoxin roles and secreted through both, the T2SS and T3SS2 (Matsuda et al., 2019). Like TDH, TRH is secreted through both, the T2SS and T3SS2 (Matsuda et al., 2019). The pathogenicity role of T3SS1 of *V. parahaemolyticus* is described below.

*Vibrio alginolyticus* express the toxic hemolysins Tdh and TLH where the former has hemolytic activity both on mouse and fish and the latter shows both hemolytic and phospholipase activities in zebrafish (Cai et al., 2007; Jia et al., 2010). *Vibrio harveyi* produce a cytotoxic hemolysin VHH with phospholipase B activity, where its native secretion system is not clear; but it has been shown to use T2SS-mediated secretion when recombinantly expressed in *E. coli* (Zhong et al., 2006; Sun et al., 2007). Several extracellular products (ECPs) from *P. damselae* sp. *damselae* that have strong hemolytic, lipase, esterase-lipase and phospholipase activity but weak proteolytic activity are reported to be lethal for fish (Fouz et al., 1993; Labella et al., 2010).

#### Secretion of Effectors

T3SSs are ubiquitous in Gram-negative pathogens including fish pathogenic *Vibrionaceae* such as *V. parahaemolyticus*, *V. salmonicida, V. alginolyticus*, *V. harveyi and Photobacterium damselae.* They directly deliver both fish and non-fish virulence factors from the pathogen cytosol into host cells where the effect of the virulence factors on fish pathogenesis varies depending on the nature of the *Vibrionaceae* members (Hueck, 1998; Frey and Origgi, 2016; Osorio, 2018).

As mentioned earlier, *V. parahaemolyticus* harbor a set of two T3SSs (T3SS1 and T3SS2) where T3SS1 is related to cytotoxicity and T3SS2 is associated with both cytotoxic and enterotoxic activities in mice (Hiyoshi et al., 2010). Several effectors such as VopQ [that induces autophagy in mammalian cell lines; (Burdette et al., 2009)], VopS [modification of host Rho family GTPases in HeLa cells (Yarbrough et al., 2009)], VopR (unknown function) and VpA0450 [hydrolyzes plasma membrane-located phosphatidylinositol phosphate in human cells (Broberg et al., 2010)] are secreted through T3SS1. Evidence based on non-fish models show that VopA/VopP [inhibits mitogen-activating protein kinase pathway using acetyl transferase, (Trosky et al., 2007; Pan et al., 2021) VopL (induce stress fiber using actin nucleastion (Ham and Orth, 2012)], VopT [Induce cytotoxicity using ADP-ribosylation (Sun and Barbieri, 2003; Barbieri and Sun, 2004)], VopV [facilitate enterotoxicity by F-actin binding/bundling (Hiyoshi et al., 2011)] and VopC [Promote bacterial invasion using deamidase (Zhang et al., 2012)] are secreted through T3SS2 (Ham and Orth, 2012). Additional contributions of T3SS2 to pathogenicity is discussed above in the section on hemolysins.

Effectors of T3SSs in *V. alginolyticus* promote cell death of fish cells (Zhao et al., 2010). Val1686 and Val168 in *V. alginolyticus*, homologous to the T3SS1 effector proteins VoPQ (88 %) and VopS (91 %) of *V. parahaemolyticus*, cause apoptosis in fish cell lines (Osorio, 2018). *V. alginolyticus* harbor two T6SSs, T6SS1 and T6SS2, where T6SS1 secretes multiple effectors such as MIX IV pore- forming effector (Va16152), MIX I (Va01565), LytM, a peptidoglycan hydrolase and lysozyme-like domain (Va01435), OmpA_C (Va01555) and VopQ (Va17542) (Salomon et al., 2015). Also *V. splendidus* produce a virulence effector, Hop, with cytotoxic effects that is secreted by a T3SS (Zhuang et al., 2020, 2021).

### Enterobacteriales

*Yersiniaceae*, *Hafniaceae*, and *Enterobacteriaceae* are three of the seven families classified under the order Enterobacteriales that contain common fish pathogens, causing mild to severe fish diseases.

*Yersinia ruckeri* is a member of the *Yersiniaceae* family, and strains from different biotypes are reported as causative agents of enteric redmouth disease (ERM). ERM, also known as yersiniosis, dominantly infects salmonids causing serious economic threats to the salmonid aquaculture industry worldwide (Austin D. A. et al., 2003; Fouz et al., 2006; Arias et al., 2007; Wheeler et al., 2009; Calvez et al., 2014; Hjeltnes et al., 2017; Wrobel et al., 2019). *Yersinia ruckeri* is a rod-shaped, nonencapsulated facultative anaerobe (Tobback et al., 2007). Typical symptoms of yersiniosis include septicemia and subsequent development of hemorrhages on the body surface and internal organs (Tobback et al., 2007). Alike some *Yersinia* species infecting other animals and humans, *Y. ruckeri* contain plasmids that encode virulence-related factors such as type IV pilus and T4SS (Wrobel et al., 2018).

*Edwardsiella tarda* [current name *E. piscicida* (Abayneh et al., 2013)] and *Edwardsiella ictaruli* belong to the genus Edwardsiella within the *Hafniaceae* family. They are economically important fish pathogens predominant in aquaculture industries worldwide (Thune et al., 1993; Mohanty and Sahoo, 2007). Edwardsiellosis is the general term used for the septicemic diseases caused by *E*. *tarda* and *E*. *ictaluri* leading to severe skin, muscle and internal organ lesions and causing high levels of mortality (Ewing et al., 1965; Abbott and Janda, 2006; Kerie et al., 2019). *Edwardsiella tarda* is a. short, mostly motile and rod-shaped facultative anaerobe that infects wide ranges of fish species (Matsuyama et al., 2005; Park et al., 2012). *Edwardsiella ictaruli* is a short and pleomorphic rod-shaped, motile species that is primarily recognized as a causative agent of enteric septicemia of catfish species. *E*. *ictaluri* was isolated from kidney and livers of several catfish with signs of enteric septicemia (Hawke et al., 1981). *Edwardsiella tarda* and *E. ictaruli* share common virulence strategies (see below).

*Citrobacter freundii* a member of the genus Citrobacter, is a long, rod shaped, facultative anaerobic bacterium of the family *Enterobacteriaceae*. Several *C. freundii* strains contain flagella used for locomotion while some are non-motile. *Citrobacter freundii* is closely related to *E.coli* and *Salmonella* and is prevalent in diverse ecological niches such as soil, water, sewage, food and the gut of animals including humans (Knirel et al., 2002; Murray et al., 2010; Abbott, 2011; Bandeira Junior et al., 2018). *Citrobacter freundii*, a causative agent of gastroenteritis and hemorrhagic septicemia, is a common pathogen of diverse freshwater fish where infected fish show symptoms such as skin ulceration, systemic infectious signs on the liver, kidney, muscles and gills (Jeremić et al., 2003; Baldissera et al., 2018; Sun et al., 2018).

#### Transmission Routes and Adherence

Host cell adherence by *Y. ruckeri* is promoted through the pathogen’s variable motility that depends on the presence and absence of flagella (Davies and Frerichs, 1989). Bacterial adherence in *Y. ruckeri* may also involve type IV pili, a hair-like multi-subunit surface appendage which its function is not restricted to surface biding and twitching motility, but also DNA-uptake, and micro-colony or biofilm formation (Mattick, 2002; Craig et al., 2019).

Experimental evidence shows that the site of entry for virulent *E. tarda* and *E*. *ictaluri* are gills, gastrointestinal tract and body surface (Ling et al., 2001; Pirarat et al., 2016). *Edwardsiella tarda* is shown to involve actin and microtubules for entry to host cells (Ling et al., 2001; Sui et al., 2017). Motility is critical during the initial phase of colonization for several aquaculture pathogens (Ormonde et al., 2000). Two types, motile and non-motile strains, of *E*. *tarda* exist where there is apparent differences in the pathogenicity between these variants (Park et al., 2012), however, the difference in colonization mechanisms of these variants are not clear. Furthermore, secretion systems such as T3SS and T6SS play pivotal roles in adherence and cell-diffusion (Tan et al., 2005; Park et al., 2012). For instance, EvpP, a component of T6SS, is shown to control the internalization process in invasion suggesting that this secretion system plays a key role in the pathogenesis of *E. tarda* (Wang X. et al., 2009).

The site of infection of *C. freundii* are the mucosal layers of the intestine which suffer severe damage during infection (Pan et al., 2021). A whole genome analysis of *C. freundii* indicates the presence of multiple virulence factors that are potentially involved in colonization and in-host cell survival. *C. freundii* displays strong adhesion to the hepatic fish cell line L8824 and can cause distinctive lesions known as A/E lesions (Pan et al., 2021), similar to A/E lesions caused by *E.coli* on the intestinal mucosa. This effect requires strong attachment of the pathogen to the surface of the enterocyte and involves a T3SS which injects multiple effector proteins into the cell (Wong et al., 2011; Hartland and Leong, 2013). An adhesin named EaeA of *C. freundii* has previously been identified as key element for colonization in mice but its role in colonization of fish is not clear (Schauer and Falkow, 1993). EaeA itself is an autotransporter, a member of the type Vc secretion systems (Leo et al., 2015).

Survival mechanisms in the hostIt is crucial for pathogens to avoid host defense mechanisms, in order to survive, proliferate and maintain infection in the host. Key to *Y. ruckeri* survival, growth and pathogenicity in the host is its iron acquisition system that produces an enterobactin-like siderophore known as ruckerbactin; the gene encoding ruckerbactin is upregulated during infection (Fernández et al., 2004; Tobback et al., 2007). The secretion and uptake systems for ruckerbactin and ferric-ruckerbactin, respectively, are not yet elucidated in detail, however, the ruckerbactin receptor has a high degree of similarity with the ferrichrysobactin receptor of *Dickeya dadantii* (Fernández et al., 2004).

*Edwardsiella tarda* has evolved several mechanisms to survive immune responses. One of these mechanisms involves the neutralization of reactive oxygen species using redox enzymes such as superoxide dismutase (SodB) and catalase KatB (Han et al., 2006). *Edwardsiella tarda* has the ability to survive in host serum, to resist acidic milieu and to replicate in phagocytes with the help of a serum-induced, putative hydrogenase protein named Sip2 (Li and Sun, 2018). In serum, the complement system has a significant role in host defense against infection via mechanisms involving both innate and adaptive immunity (Walport, 2001; Merle et al., 2015). Activation of the complement system lead to bacterial membrane damage and subsequent lyses using membrane attack complex (MAC) (Endo et al., 2006; Sarma and Ward, 2011). *Edwardsiella tarda* has the ability to evade the bactericidal effect of host serum through blocking the activation of the complement using the zinc metalloprotease Sip1 (Zhou et al., 2015). Besides, serum enhances the tricarboxylic acid cycle of *E*. *tarda* which increases membrane potential and decreases the formation of MAC, leading to serum resistance (Cheng et al., 2017). Furthermore, the chaperone protein HtpG is shown to help *E. tarda* to cope with stress conditions during infection (Dang et al., 2011).

Like *E. tarda*, *E*. *ictaluri* has the ability to survive and grow within host macrophages (Miyazaki and Plumb, 1985; Baldwin and Newton, 1993; Pirarat et al., 2016). A T3SS translocates several effectors from *E*. *tarda* and *E*. *ictaluri* directly into the host cytoplasm, where they have essential roles for internal replication and virulence (Dubytska et al., 2016). The T3SS of *E. tarda* plays important roles in phagocyte survival, in host proliferation and virulence (Tan et al., 2005). For instance, the T3SS effector EseJ facilitates cell proliferation in host cells by inhibiting the oxidative stress produced by host macrophages (Xie et al., 2015). Like the T3SS, a T6SS has a pivotal role in survival and replication of *E*. *tarda* in host epithelial cells and phagocytes (Park et al., 2012). However, the mechanisms of the two secretion systems are antagonistic, where the T3SS promotes bacterial replication in host cells while the T6SS protects the pathogens from attack by the activated innate immune system through suppressing replication (Xie et al., 2015; Hu et al., 2019).

Iron acquisition systems such as siderophore biosynthesis and iron uptake systems are necessary for pathogenicity of *E. tarda* (Castro et al., 2016). The genome of *E*. *tard*a contains genes encoding for multiple factors putatively involved in iron utilization including ferric uptake regulator, ferric reductase, ferritin and TonB (Wang Q. et al., 2009). It also includes a gene cluster sharing high similarity to the *pvsABCDE-psuA-pvuA* operon which encodes proteins for synthesis and utilization of vibrioferrin siderophore of the two fish pathogen vibrios *V*. *parahaemolyticus* and *V*. *alginolyticus* (Tanabe et al., 2003; Wang Q. et al., 2007).

In contrary, there are no dedicated siderophore biosynthesis genes detected in the genome of *E*. *ictaluri* (Santander et al., 2012), however, the pathogen carries a ferric hydroxamate uptake (Fhu) system involved in uptake of hydroxamate-type siderophores. Experimental evidence shows that the Fhu-system is indeed involved in pathogenicity, suggesting that the system may be utilized to uptake hydroxamates secreted by other bacteria (Abdelhamed et al., 2016). In addition, the genome of *E*. *ictaluri* contains an alternative ferric uptake system (afuABC) and the general TonB energy transducing system (TonB-ExbB-ExbD) which may be involved in iron uptake (Abdelhamed et al., 2013, 2017). Furthermore, *E*. *ictaluri* utilizes a Fur-regulated heme-hemoglobin uptake system (Santander et al., 2012).

In addition to the siderophore-dependent systems, *E. tarda* can utilize hemin, hemoglobin and hematin as iron sources (Abbott and Janda, 2006). A heme utilization operon in its genome that encodes for three proteins identified as HutW_*Et*_, HutX_*Et*_ and HutZ_*Et*_ is highly similar to the heme utilization operon in *V. cholera* (Shi et al., 2019). In *V. cholera* HutX_*Vc*_ functions as an electron carrier to the heme-degrading HutZ_*Vc*_ while HutW_*Vc*_ may be a reductase for HuyZ_*Vc*_ (Sekine et al., 2016). HutZ_*Et*_ has been shown to be involved in biofilm formation and motility (Shi et al., 2019). Furthermore, *E. tarda* produce an iron-mediated hemolysin that is released under iron-limited conditions (Janda and Abbott, 1993; Hirono et al., 1997). All in all, iron depletion in host cells is a signal for pathogens to turn on the expression of virulence genes. The ferric uptake regulator (Fur) protein senses iron depletion in, for example, *E*. *tarda* to regulate expression of key virulence factors such as T3SS and T6SS through secretion regulator protein EsrC (Chakraborty et al., 2011).

*Citrobacter freundii* harbor genes that could be utilized in diverse mechanisms and virulence factors to maintain infection, including a T3SS that plays a crucial role in the injection of effector proteins such as HopAJ2 family member that mediate survival against host immune response (Galán et al., 2014; Pan et al., 2021). Iron acquisition in *C. freundii* is facilitated by FepE, a component of the ferric enterobactin siderophore transport system (Wang et al., 2019). In addition to the siderophore-dependent iron transport system, an hemophore-mediated heme uptake apparatus is present that includes an outer membrane channel protein HasF (Létoffé et al., 2001). In addition, surface lipopolysaccharides (LPS) of some serogroups of *C. freundii* have been shown to play vital roles in colonization and host survival (Denkin and Nelson, 1999).

#### Proteases and Their Secretion Systems

*Yersinia ruckeri* produce a serralysin metalloprotease named Yrp1 that is secreted via an ATP-dependent T1SS composed of three genes, *yrpD*-*F*, and a protease inhibitor *inh*. Yip1 seems to have pivotal roles in the development of ERM due to its ability to disrupt several extracellular matrix proteins of fish such as laminin and fibronectin, and proteins important in muscle function such as actin and myosin (Fernández et al., 2002). Furthermore, *Y. ruckeri* produce two additional peptidases, YrpA and YrpB encoded by the *yrpAB* operon. Toxicity tests indicate that at least YrpA is involved in *Y. ruckeri* pathogenicity, as deletion of the gene encoding YrpA drastically reduces the virulence of the bacteria (Navais et al., 2014).

*Edwardsiella tarda* produce multiple virulence-related proteases that are engaged in diverse processes of infection. For instance, the above mentioned zinc metalloprotease Sip1 of *E. tarda* is secreted to effectively prevent complement-mediated serum killing through blocking the activation of the complement system (Zhou et al., 2015). A serine protease autotransporter Tsh_*ET*_, which is a temperature-sensitive hemagglutinin, belongs to the Type V secretion systems (T5SS) and contributes to the virulence of *E. tarda*. Tsh has multiple functions; mutations in the *tsh* gene led to hindrance of biofilm growth, reduction of resistance against serum killing, impairment of the ability to block the host immune response, and attenuation of tissue invasion and cellular infectivity (Hu et al., 2016). In addition, the periplasmic serine protease DegP_*ET*_ is presumably involved in virulence of *E. tarda*, similar to DegP in *Salmonella enterica*, *Streptococcus pyogenes* and *Legionella* (Johnson et al., 1991; Jones et al., 2001, 2002; Pedersen et al., 2001; Wilson et al., 2006; Flannagan et al., 2007; Jiao et al., 2010).

#### Hemolysins and Their Secretion Systems

The gene cluster *yhlBA* of *Y. ruckeri* encodes for a hemolysin (YhlA) and a protein involved in its secretion/activation (YhlB). The expression of YhlA is upregulated in iron-limited conditions suggesting that the hemolysin may be directly involved in acquisition of iron from the host cell (Fernández et al., 2007; Wrobel et al., 2019). Both *yhlA* and *yhlB* genes have high sequence similarity with a type Vb secretion system with hemolysin activity in *Serratia* sp. (Wrobel et al., 2019).

*Edwardsiella tarda* produce two different hemolysins, the iron-mediated cell associated hemolysin EthA, secreted under iron limited conditions, and an extracellular pore-forming hemolysin district from EthA (Janda and Abbott, 1993; Chen et al., 1996; Hirono et al., 1997). Similar to the hemolysin YhlA of *Y. ruckeri* and its activation/secretion protein YhlB (described above), the hemolysin EthA from *E. tarda* needs an activation/secretion protein EthB encoded in an operon. EthA and EthB, are prevalent in hemolytic *E. tarda* strains isolated from diseased fish (Hirono et al., 1997) which may indicate that they are among the key virulence mechanisms utilized by the pathogenic *E. tarda*.

#### Polysaccharide Degrading Enzymes

Chondroitinases are involved in the pathogenicity of infectious bacteria (Schaechter et al., 1993). Chondroitinase activity is known to be virulence factor in *Edwardsiella* spp. including *E. tarda* and *E. ictaruli.* These species produce chondroitinase that mediates cartilage degradation in the process of invasion (Waltman et al., 1986; Shotts and Cooper, 1996). The Sialidase NanA from *E. tarda* also promotes tissue invasion, and mutations of the gene encoding for the enzyme led to drastic attenuation of the pathogen, by limiting its ability for invasion and colonization (Jin et al., 2012).

#### Secretion of Other Toxin Proteins or Toxins and Their Secretion Pathway

Distinct strains of *Y. ruckeri* produce TcpA, a Toll/interleukin-1 (TIR) domain containing protein that inhibits Toll-like receptor signaling and promotes immune evasion. TcpA is also known to increase tissue damage in fish. The gene for TcpA is located adjacent to a T4SS gene cluster which may indicate that the secretion of TcpA is related to this T4SS (Liu et al., 2019). As discussed above, T3SS are a crucial and multipurpose system in *E. tarda* and *E. ictaluri*. The T3SS of *E. tarda* belongs to the Ssa-Esc family which includes T3SS encoded by Salmonella pathogenicity island 2 (SPI-2) in *Salmonella enterica* serover Typhimurium, the causative agent of foodborne illness worldwide (Anderson and Kendall, 2017). The core components of the T3SS are encoded by 34 genes, where all obtain different functions (Tan et al., 2005; Sadarangani et al., 2013). Among these are the three translocon proteins EseB, EseC and EseD that are essential for delivery of effectors into host cells (Tan et al., 2005).

### Aeromonadales

The order Aeromonadales contains only two families, the *Succinivibrionaceae* and the *Aeromonadaceae.* Species of the family *Aeromonadaceae* are known to cause acute hemorrhagic septicemia in fish (Colwell et al., 1986; Stackebrandt and Hespell, 2006). *Aeromonas* species, ubiquitous in aquatic environments, are classified into two groups; motile mesophilic aeromonads with optimal growth temperature around 37°C, and non-motile psychrophilic strains with an optimal growth temperature range of 22–28°C where the latter are cold-water fish pathogens. *Aeromonas bestiarum*, *Aeromonas sobria*, *Aeromonas hydrophila* and *Aeromonas veronii*, all categorized as motile and mesophilic, are responsible for motile *Aeromonas* septicemia (MAS), an acute hemorrhagic septicemia, or for chronic skin ulcers (Ali, 1996; Stratev and Odeyemi, 2017). *Aeromonas salmonicida* comprising both non-motile psychrophilic and motile mesophilic strains is a causative agent for systemic furunculosis, a disease that causes sepsis, hemorrhages, muscle lesions, inflammation of the lower intestine, spleen enlargement, and death in freshwater fish populations (Beaz-Hidalgo and Figueras, 2013). Genome information of diverse *Aeromonas* species is available and indicates specialized pathogenic mechanisms (Seshadri et al., 2006; Reith et al., 2008; Li et al., 2011; Beaz-Hidalgo and Figueras, 2013). Virulence by *Aeromonas* is a complex succession of processes, where successful infection requires potential for the formation of biofilms, production and secretion of virulence factors such as adhesins, proteases, hemolysins, lipases, DNases and effector proteins as well as regulation of virulence factors through quorum sensing (Allan and Stevenson, 1981; Cahill, 1990; Thornley et al., 1997; Beaz-Hidalgo and Figueras, 2013).

#### Transmission Routes and Adherence

The important initial infection sites for *Aeromonas* such as *A. salmonicida* include skin, gills and the gastrointestinal tract (Ringø et al., 2004; Bartkova et al., 2017). Tissue invasion in the gastrointestinal tract is mediated by extracellular virulence factors that are utilized to damage the tissue (Ringø et al., 2004). Adhesion, a prerequisite for successful colonization by pathogens, is executed by different factors in *Aeromonas species*. Outer membrane proteins such as OmpA and AHA1 function as adhesins in *A. veronii* and *A. hydrophila* (Fang et al., 2004; Namba et al., 2008). A tight adherence system (TaD) is present in the majority of *A. hydrophila* strains (Tekedar et al., 2019a). *A. hydrophila* and *A. sobria* use pili (Lallier and Daigneault, 1984; Ho et al., 1990), lipopolysaccharides (LPS) is used by *A. hydrophila* (Merino et al., 1996), and cell-associated lectins are used by *A. veronii* (Guzman-Murillo et al., 2000) to bind to host cells and tissues. Paracrystalline surface protein layers in *A. salmonicida* and *A. hydrophila* display significant hydrophobicity and contribute to adhesion (Chu et al., 1991; Beveridge et al., 1997).

#### Survival Mechanism in the Host

Survival of *A. salmonicida* and *A. hydrophila* in the host is achieved by S-layers that bind to extracellular matrix components such as laminin, fibronectin and vibronectin providing resistance to serum killing and protease digestion (Beveridge et al., 1997; Noonan and Trust, 1997). Most *Aeromonas* sequester iron from their hosts through utilization of siderophore-dependent or siderophore-independent (heme-binding) mechanisms (Byers et al., 1991; Reith et al., 2008; Lange et al., 2020). The siderophore-dependent mechanisms rely on the synthesis of enterobactin or amonabactin and some *A. salmonicida* species are capable of synthesizing anguibactin-like siderophores (Barghouthi et al., 1989a,b; Byers et al., 1991; Telford and Raymond, 1998; Reith et al., 2008). *Allivibrio salmonicida* and *A. hydrophila* synthesize both ferric and heme iron acquisition systems (Ishiguro et al., 1986; Najimi et al., 2008b). In most cases, two catechol siderophores; acinetobactin and amonabactin, are produced simultaneously (Balado et al., 2015) and their uptake is facilitated by the TonB-dependent outer membrane proteins FstB and FstC that function as receptors for ferric-acinetobactin and ferric-amonabactin, respectively (Balado et al., 2017; Rey-Varela et al., 2019). Ferric-siderophore uptake is mediated by FstA in some species of *A. bestiarum* and *A. salmonicida* (Beaz-Hidalgo et al., 2008, 2013). An multifunctional amonabactin receptor of *A. hydrophila* is reported to transport several different siderophores across the membrane (Stintzi and Raymond, 2000). Heme uptake is performed by the membrane receptors HgpB in *A. veronii* and HutA in *A. salmonicida* and *A. hydrophila*, (Najimi et al., 2008a; Maltz et al., 2015).

#### Virulence Factors and Their Secretion Systems

*Aeromonas* utilize several secretion systems such as the sec-dependent systems T1SS, T2SS, and the sec independent T3SS and T6SS to transport virulence factors, toxins and effectors for infecting diverse fish species (Burr et al., 2002; Tekedar et al., 2019b). The T1SS and T2SS are prominent in almost all *Aeromonas* species, and a T3SS is detected in species such as *A. salmonicida*, *A. hydrophila*, and *A. veronii* (Citterio and Biavasco, 2015; Tekedar et al., 2019b; Barger et al., 2020). A T6SS was detected for the first time in the genomes of *A. hydrophila* and *A. salmonicida* (Seshadri et al., 2006; Reith et al., 2008).

#### Proteases and Their Secretion Systems

Proteases are among the main virulence factors in *Aeromonas* (Sakai, 1985). Three types of proteases, metalloproteases, acetylcholinesterases and serineproteases are produced by *Aeromonas* (Seshadri et al., 2006). Both metallo- and serine proteases are secreted trough a T2SS by *A. hydrophila* (Sandkvist, 2001). A serine protease (AspA) as well as a glycerophospholipid:cholesterol acetyltransferase (GCAT; a lipase; see below) are secreted by *A. salmonicida* and were previously thought to be main virulence determinants (Buckley et al., 1982; Lee and Ellis, 1990; Whitby et al., 1992; Coleman and Whitby, 1993; Bernoth et al., 1997), however, it has later been shown that their deletion has minor effects on pathogenicity (Vipond et al., 1998).

#### Hemolysins and Their Secretion Systems

The presence of hemolytic capability in *Aeromonas* is associated with the development of lesions and mortality in fish species such as rainbow trout (Ellis et al., 1988). *Aeromonas* species such as *A. hydrophila* produce α-hemolysin (HlyA), a pivotal virulence factor causing cell-rounding and apoptosis (Tekedar et al., 2019a). HlyA is an Rtx-type toxin that are known to be secreted through T1SS in *Vibrio* species (Boardman and Satchell, 2004). Other pore-forming Hemolysins including H-lysin, T-lysin, salmolysin, ASH1, ASH3 and ASH4 are produced by *A. salmonicida* to lyse erythrocytes (Titball and Munn, 1981, 1985; Nomura et al., 1988; Hirono and Aoki, 1993). Secretion mechanisms of most of the hemolysins discussed here are not yet investigated. However, mutation of a T2SS in *A*. *hydrophila* led to impaired hemolytic activity suggesting that the T2SS may be one of a secretion mechanism for at least some hemolysins in some members of the *Aeromonas* species (Barger et al., 2020). Aerolysin, a hemolysin, is a cytolytic pore-forming toxin produced by *A. hydrophila*, *A. salmonicida* and *A. sobria* (Chakraborty et al., 1990; Wilmsen et al., 1990; Hirono and Aoki, 1993; Fivaz et al., 2001) which binds to host cells and leads to increased membrane permeability. Aerolysin from *A. hydrophila* is known to be secreted through a T2SS (Ast et al., 2002).

#### Lipases and Their Secretion Systems

Glycerophospholipid:cholesterol acetyltransferase (GCAT) is an unusual lipase involved in the pathogenicity of *A. salmonicida* (Lee and Ellis, 1990). Combinations of GCAT and lipopolysaccharide (LPS) from *A. salmonicida* have lethal effects on salmon and rainbow trout (Røsjø et al., 1993; Lachmann et al., 1998). Glycerophospholipid:cholesterol acetyltransferase is secreted through T2SS (Sandkvist, 2001).

#### Elastase and Their Secretion Systems

The role of elastases from numerous bacterial pathogens as virulence factor is well documented (Kamath et al., 1998; Cascón et al., 2000; Bleves et al., 2010; Li J. et al., 2019). Among these, an extracellular elastase AhyB produced by *A. hydrophila* has high elastolytic activity in non-fish model and is essential for pathogenicity (Cascón et al., 2000). AhyB is secreted through T2SS (Cascón et al., 2000; Barger et al., 2020).

#### DNase and Their Secretion Systems

A DNase produced and secreted through a T2SS is present in the genome of *A. hydrophila* (Sandkvist, 2001), which is not yet fully characterized but is predicted to have an impact in virulence.

#### Toxins and Their Secretion Systems

An extracellular ADP-ribosylating toxin (AexT) is produced by *A. salmonicida*, similar to the mechanism of Exoenzyme S (ExoS) in *Pseudomonas aeruginosa* (Yahr et al., 1996a,b) to modify host cell proteins hence induce disease. One example of this is AexT from *A. salmonicida* (Burr et al., 2003; Dacanay et al., 2006) where experimental evidence confirmed that this ADP-ribosylating toxin is directly translocated into the host cytosol by T3SS, similar to its *P. aeruginosa* homolog ExoS (Yahr et al., 1996a,b; Braun et al., 2002; Dacanay et al., 2006; Silver and Graf, 2009).

#### Effectors and Their Secretion Systems

T3SSs are generally considered as a key virulence factor in many bacterial pathogens including *Aeromonas* due to their needle structure enabling injection of specific toxins directly into the host cytosol (Vilches et al., 2004; Dacanay et al., 2006; Coburn et al., 2007; Fast et al., 2009; Austin and Austin, 2016). Several T3SS effectors of *Aeromonas* are reported to affect the host immune response. AopH and AopO are effectors secreted by *A. salmonicida* that are secreted via the T3SS and that are highly similar to the effector proteins YopH and YopO from *Yersinia* species that influence cytoskeleton functions. YopH dephosphorylates tyrosines in focal adhesion proteins while YopO modulate the function of Rho GTPase to impair its roles in gene transcription, regulate actin cytoskeleton, control cell cycle and intracellular vesicle transport (Aepfelbacher, 2004). AopP is injected directly into host cells via T3SS to influence the host inflammatory response through inhibiting the nuclear factor- κB (NF-κB) signaling pathway (Fehr et al., 2006; Frey and Origgi, 2016). Other T3SS effector proteins such as the inositol polyphosphate 5-phosphatase Ati2 (Dallaire-Dufresne et al., 2013), HrpJ or BopN-like effector protein (AopN) (Nagamatsu et al., 2009; Crabill et al., 2012; Bergh and Frey, 2014), and ExsE of *A. salmonicida* have been shown to down-regulate host inflammatory responses (Bergh and Frey, 2014). In contrast to this, Aerolysin produced by *A. hydrophila* is secreted by a T2SS (Sandkvist, 2001).

The T3SS in *A. salmonicida* can be lost through genome modifications triggered by mobile elements such as insertion sequence (IS) elements when the bacteria are grown at temperatures exceeding 25°C, changing the strain phenotype from virulent to non-virulent (Tanaka et al., 2012, 2017). Whole genome analysis of 33 strains of *A. hydrophila* indicate the presence of T3SS, and mutation of two of the T3SS genes *aopB* (a translator) and *aopD* (a transmembrane protein) decreases cytotoxicity in carp epithelial cells, reduces virulence in blue gourami and increases phagocytosis (Yu et al., 2004). However, some hyper-virulent strains of *A. hydrophila* lack core components of the T3SS, which may indicate that these pathogens have an alternative secretion mechanism for virulence factors (Hossain et al., 2013; Pang et al., 2015).

### Others Fish Pathogens From the Proteobacteria

#### Thiotrichales

*Piscirickettsia salmonis*, a member of Piscirickettsiaceae family, genus Piscirickettsia is the causative agent of piscirickettsiosis, also known as salmon rickettsial syndrome (SRS), a disease with high mortality that affects several seawater fish species. *P. salmonis* is non-motile, aerobic, encapsulated and pleomorphic (Fryer et al., 1990, 1992). *Piscirickettsia salmonis* is an intracellular pathogen causing remarkable economic loss in the aquaculture industry worldwide (Rozas and Enríquez, 2014; Figueroa et al., 2019). Diseased fish are dark in color, anemic where their kidney is swollen and the liver develops lesions (Fryer et al., 1992).

#### Transmission Routes and Adherence

The main routes of entry of *P. salmonis* in rainbow trout is through skin and gills (Smith et al., 1999). Studies on *P. salmonis* adherence and mode of invasion using fish eggs show that the pathogen attaches to the ova by means of membrane extension which allows penetration into the cell (Larenas et al., 2003). *P. salmonis* replicates within membrane-bound cytoplasmic vacuoles in host cells (Fryer et al., 1992). In stress conditions, *P. salmonis* form a biofilm-like cell aggregate that disintegrates when treated with cellulase, which indicates the presence of polysaccharides that are common for biofilm formation (Marshall et al., 2012). In addition, some lectins show strong binding to the biofilm-like structure, which confirms the presence of an exopolysaccharide (Rozas and Enríquez, 2014).

*Piscirickettsia salmonis* is capable of growing and surviving in fish macrophages where the bacteria are partially enclosed into vacuole membrane vesicles which escape destruction within phagolysosomes (Almendras and Fuentealba, 1997; McCarthy et al., 2005, 2008). *Piscirickettsia salmonis* possess genes belonging to the Dot/Icm-type IV secretion system (T4SS) which is a type of secretion system utilized as major virulence mechanism in related intracellular pathogens such as *Legionella pneumophila* and *Coxiella burnetii* where the system is used for intracellular survival and replication (Thomas et al., 2020). It is shown that *P. salmonis*-containing vacuoles do not fuse with lysosomes, which suggests the presence of bacteria-mediated interference with the endosomal maturation process to ensure bacterial survival. This process depends on the Dot/Icm-type IV secretion system that delivers effector proteins into the host cytosol (Rozas and Enríquez, 2014; Zúñiga et al., 2020). As an additional virulence factor, *P. salmonis* contains lipopolysaccharides with endotoxin activity (Cvitanich et al., 1991). Moreover, *P. salmonis-*infected fish show a downregulation of genes involved in the adaptive immune response (Tacchi et al., 2011).

The molecular virulence mechanisms of *P. salmonis* are poorly described. However, it is known that extracellular extracts of *P. salmonis* contain products with cytotoxic and exotoxin effects (Rojas et al., 2013). Outer membrane vesicles containing several outer membrane proteins are released by *P. salmonis* during intracellular replication, including OmpA, which is involved in biofilm formation, adherence or invasion in other species. The chaperonin Hsep60, which is a cytoplasmic component but has been shown to be secreted by some pathogenic bacteria (Garduño et al., 1998; González-López et al., 2013) is presumably involved in adherence to host cell membranes (Ensgraber and Loos, 1992; Huesca et al., 1996). The chaperone HtpG that is involved in pathogenesis of *E. tarda* (Dang et al., 2011) is detected in the vacuoles during *P. salmonis* infections (Oliver et al., 2016).

Genome analysis of *P. salmonis* shows the presence of several other potential virulence factors (Ortiz-Severín et al., 2019). These include a Phospholipase D-like domain protein similar to the toxin Ymt from *Yersinia pestis* (Otsuka, 2016); three PipB2 pentapeptide repeate-containing proteins that potentially contribute to replication in intracellular vesicles as described for their homologs in *Salmonella enterica* (Knodler and Steele-Mortimer, 2005), an ATPase related to the flhG ATPase of *Campylobacter fetus*, which is part of the flagellar apparatus (Henderson et al., 2020), and a glutamate-1-semialdehyde-2,1-aminomutase whose *Haemophilus somnus* homolog is incolved in heme-dependent iron uptake (Villarreal, 2008).

#### Alteromonadales

*Moritella viscosa*, a member of *Moritellaceae* family, is a rod-shaped psychotrophic bacterium that is a main causative agent of winter ulcer, a cold season disease that infects a wide variety of salmonid fish in sea water. Diseased fish shown swelled skin, and develop lesions, hemorrhages and tissue necrosis. The mortality rate of winter ulcer is relatively low but the disease represents a significant fish welfare problem (Bruno et al., 1998; Løvoll et al., 2009).

Adherence of *M. viscosa* to various mucosal surface has been reported (Tunsjø et al., 2009). Gills are reported as the site of entry of *M. viscosa* from where the pathogens penetrate further into muscles, kidney, spleen and liver, a sign that the pathogen can cause systemic infections (Løvoll et al., 2009). The genome of *M. viscosa* comprises both flagella and type IV pili system genes. Adherence to fish cells is facilitated by flagella and higher level of adherence is recoded at low temperature. During adhesion by *M. viscosa*, aggregation of F-actin microfilaments of the cell line CHSE was observed and the host cell membrane disrupted (Tunsjø et al., 2009).

*Moritella viscosa* seem to survive in the host using escape mechanisms that suppress immune responses and help to evade the host immune system (Løvoll et al., 2009). *Moritella viscosa* contains lipopolysaccharides (LPS) and other outer membrane antigens with potential to protect the pathogen in host cells (Heidarsdottir et al., 2008). *Moritella viscosa* uses a metallopeptidase, MvP1 (see below for details) that is involved in invasion and dispersion of the pathogen (Bjornsdottir et al., 2009).

The virulence mechanisms of *M. viscosa* have not been extensively studied. The extracellular products (ECP) of the pathogen contain several secreted virulence factors with both cytotoxic and hemolytic activities that lead to the disease phenotype in Atlantic salmon (Bjornsdottir et al., 2009). The ECP contains enzymes and toxins (Benediktsdóttir and Heidarsdóttir, 2007). Genome analysis of *M. viscosa* shows the presence of three types of secretion systems: T1SS, T2SS and T6SS. In addition, vesicle-like structures have been observed by electro-microscopy (Tunsjø et al., 2009; Bjornsdottir et al., 2012).

The metallopeptidase MvP1 is an extracellular protease secreted by *M. viscosa*, formerly identified as vibrolysin with virulence related activities. By itself it is non-lethal to salmon at low concentrations. But MvP1 causes severe hemorrhages and degrades host tissues leading to necrosis, and affecting adhesion. MvP1 is suggested to be involved in invasion and dispersion in the host (Bjornsdottir et al., 2009; Björnsdóttir, 2011).

Virulence-related genes detected in several *M. viscosa* strains include the T6SS ATPase (clpV) and hemolysis co-regulated proteins (hcp) (Bjornsdottir et al., 2012) as well as Bacterioferritin homologs, which are known to enhance *Pseudomonas putida* survival in iron depleted environments (Chen et al., 2010). Further examples include a Hemagglutinin (hemG), a lectin that is produced during cell aggregation (Romeo et al., 1986) and a multifunctional autoprocessing repeats-in-toxin (martxA) from the RTX family of toxins that are secreted via a T1SS (Satchell, 2007).

#### Pseudomonadales

*Pseudomonas anguilliseptica*, is a rod-shaped and flagellated member of the *Pseudomonadaceae* family (Palleroni, 2010). *Pseudomonas anguilliseptica* is a known pathogen and a serious threat to the production of a variety of fish species cultured in marine and brackish water in different parts of the world (Stewart et al., 1983; Wiklund and Bylund, 1990; Toranzo and Barja, 1993; Lönnström et al., 1994; Berthe et al., 1995; Fernández et al., 2002; Austin and Austin, 2016). The pathogen causes a disease named “red spot disease” also known as “sekiten-byo,” a hemorrhagic septicemia that was first discovered in Japan (Wakabayashi and Egusa, 1972). The disease caused by *P*. *anguilliseptica* is sometimes associated to winter disease; however, this is considered as misrepresentation as some authors consider winter disease as multifactorial condition linked with cold and stressful environments (Tort et al., 1998; Contessi et al., 2006).

Despite the seriousness of the threat of *P. anguilliseptica* to the aquaculture industry worldwide, knowledge on virulence mechanisms of this pathogen is scarce. The site of entry for *P. anguilliseptica* is not clear, but the presence of flagella indicates that the pathogen has the capacity for motility and host cell adhesion (Berthe et al., 1995). *P. anguilliseptica* possesses capsular (K) antigens, which was first observed in a case of eel infection. This antigen may be used for escaping complement mediated killing by fish serum (Nakai, 1985; López-Romalde et al., 2003b).

## Discussion

This review can only give a glimpse on the many variations of protein secretion in commercially relevant fish pathogens. Molecular information is extremely scarce in many cases, despite the importance of the diseases caused. Part of this problem is that in most cases, the researchers working with fish diseases by tradition are not molecular biologists, but veterinarians more interested in the treatment of acute conditions using traditional antimicrobial therapies. Vaccine development in the aquaculture industry is also not based on molecular targets and studies, but rather relies on heat-killed bacterial extracts for cost reasons. The information is even more scarce for Gram-positive fish pathogens, where almost all molecular information on virulence mechanisms is only inferred from closely related human-pathogenic species. This is especially true for fish diseases with very broad and general symptoms that are hard to connect to a single pathogenic species or strain, such as streptococcosis, that is caused by diverse *Streptococcus* species depending on the host fish (Buller, 2004). A notable exception is *Mycobacterium marinum*, the causative agent of fish tuberculosis, that has received a lot of attention because it infects the model species zebrafish and thus makes a formidable model system for the study of human tuberculosis disease mechanisms (Bouz and Al Hasawi, 2018).

We strongly believe that a better molecular understanding of protein secretion in fish pathogens can help to develop more targeted therapies for the aquaculture industry, with the aim to avoid the use of antibiotics in aquatic habitats, and hope that this overview helps interested researchers in their quest to develop new vaccines or drugs.

## Author Contributions

SM wrote the manuscript. DL edited the manuscript. Both authors contributed to the article and approved the submitted version.

## Conflict of Interest

The authors declare that the research was conducted in the absence of any commercial or financial relationships that could be construed as a potential conflict of interest.

## Publisher’s Note

All claims expressed in this article are solely those of the authors and do not necessarily represent those of their affiliated organizations, or those of the publisher, the editors and the reviewers. Any product that may be evaluated in this article, or claim that may be made by its manufacturer, is not guaranteed or endorsed by the publisher.
